# Odontological Society of Pennsylvania

**Published:** 1896-01

**Authors:** 

**Affiliations:** Odontological Society of Pennsylvania


### ODONTOLOGICAL SOCIETY OF PENNSYLVANIA.

A MEETING of the Odontological Society of Pennsylvania was held Saturday, November 9, 1895, at 1415 Walnut Street, Philadelphia.

The paper of the evening, "The Heroic in Dental Operation, as contrasted with Conservatism," was read by Dr. Kratzer.

(For Dr. Kratzer's paper, see Volume XVI., page 744.)

### DISCUSSION.

*Dr. Peirce.*—I think Dr. Kratzer strikes the key note when he says that we ought to work for our patients' benefit rather than our own glory. I remember some few years ago a lady coming from a neighboring city. She told me she had paid six hundred dollars for having her teeth contoured, and she had not had a comfortable day since, although she had tolerated the work for several years. It was beautifully done, but on frail teeth with roots predisposed to irritation, and while there were no suppurative processes, yet the gums were tender. There were two bicuspids that especially troubled her, and she requested some relief. I took out the fillings and cleansed the devitalized roots. The teeth had been wedged apart previously to contouring, and had not been allowed to return to their normal position. The fillings extended over the faces of the roots so that there was constant impingement of the crowns, one upon the other. She said she did not mind the appearance of gold, so the roots were supplied with gold caps, and these she is wearing comfortably to-day.

It is a mistake to keep patients in the chair four, five, or six hours, while malleting a gold filling, when we could make a better operation by trimming off the tooth properly and adjusting a collar and a porcelain crown.

One of my patients said, I don't see any necessity for having my teeth fixed, if I am to suffer a penalty equal to death, and she was correct in this opinion. There are dentists who delight in large contour fillings, which so exhaust the patients that they oftentimes are in bed for days.

Where we have an approximal surface of a tooth to be restored, it should be contoured carefully. But where half or two-thirds of the crown is gone it is unjust to the patient to build it up piece by piece.

*Dr. Faught.*—The day for the heroic in dentistry is passed

There are times when it may be good to indulge in these protracted operations, but when we do that kind of work it is only as a necessity. The more we shape our practice to the conservation of the teeth by the easier and less protracted operations, the better it will be for our patients and the better for us individually.

My own practice is made up to some extent of nervous people. Many times I have seen an opportunity for building up large fillings, but I have adopted other methods.

Particularly do I believe the conservative method of the greatest value where we have to deal with children. I remember a case in my practice where I had a family of three or four boys over a period of three years, and just this winter am I successful in placing all their mouths in perfect condition, but it was only by doing conservative work while they were young that I was able at the proper time to replace plastics with gold.

I will recite a case that left a profound impression on me. It was with a family of three children,—nervous, growing girls. These children came to me, and by the use of gutta-percha against the frail enamel walls I was able to make them comfortable, and to bring their teeth into the condition where later on I would be able to execute more lasting and permanent gold work.

One year, just as the summer months were approaching, I failed to see one daughter. She was away at boarding-school. The mother said she thought she would have to excuse her from seeing me until after vacation. I warned her that there was a cavity that ought to be attended to, and that in a few weeks I would be closing the office and going away on my summer trip. The patient did not come in. During the time I was away the father took this daughter to his dentist, who not only filled this cavity, but suggested the removal of the gutta-percha fillings, and also suggested that there had been a good deal of very poor dentistry executed there. It was summer time, when our visits are not as numerous as they might be, and he ruthlessly cut away the enamel. The teeth were so sensitive and tender that he was obliged to etherize her in order to excavate the cavities and prepare for the insertion of the filling. She spent two or three hours in the chair each sitting, and in going out on one occasion fainted at the corner of the street.

He not only did that, but went further and suggested that perhaps the other daughters' mouths had been under the care of the same gentleman and were in the same condition. The father, very much exercised, took the other children there and had them treated in the same way. The result was that not one day elapsed that those children were not in agony; every mouthful of hot or cold food in the oral cavity brought tortures and pangs, and they never had one moment of peace from the day they sat in the chair until in the fall, when they came to me again, brought back by the mother, who desired that I should take away the heroic operations and replace them with similar gutta-percha fillings to those that were there before. I could not do it, of course; the enamel walls had been cut away to the very edge; but I did the best I could. The result was that the pulps died, and the mouths now are wrecks in comparison with what they might have been had conservative dentistry been allowed full sway.

There is another thing that I have been very much interested in. There is a little preparation in the market to-day under the name of Power's antiseptic balsam, and I have been giving it a trial, with much satisfaction. It is used by touching it to the cervical edge of the cavity, without cutting retaining pits. It is easy to attach the first piece of gold to the walls, and thus excellent and desirable gold fillings can be made without subjecting the patient to the pain or the detriment of cutting away the cervical edge, and leaving the enamel without dentine back of it. Care is to be exercised to place it well back within the cavity.

*Dr. Jamison.*—I cannot say that I entirely agree with the former speaker. I have not been a great advocate of plastics; I rather incline to the gold theory where it can be properly used. As we get older we become more conservative. The majority of the young men who come from our colleges are apt to be heroic; very apt to delight in large gold fillings and contours, and I think, in a measure, it is good that they do, if the patients are not made uncomfortable. I am not an extremist. The key to the whole subject is judgment; this should be exercised, and that covers the whole ground.

*Dr. Boice.*—While the paper was being read my mind reverted to the case of two sisters who did not dare to come to the office alone. They had in previous operations required the attention of a physician after they had been to the dentist, but after I had had charge of them for about a year they no longer feared to come alone.

I met a gentleman on Chestnut Street one day, and he said, "By the way, won't you make an appoinment for my wife? She has lost another of her fifty dollar fillings." I did so and filled the cavity in twenty minutes with amalgam. The original filling had required seven hours and she was in her room for one week. The gold filling lasted four years and the amalgam has been there for seven.

The gentleman who put the fillings in, I think I can say, has been one of the experts in packing gold in the city of Philadelphia. He believed in the heroic treatment. He said he never filled with amalgam; he despised gutta-percha.

I have long ago abandoned the idea that it was my duty to punish patients by keeping them in the chair three, four, or five hours to build up an old root with gold when I could put a crown upon it that would be more serviceable.

The essayist suggested something about sea-sand and a cantilever bridge. The mass of crown- and bridge-work has been appalling. To-day I examined a crown on a bicuspid, the band of which was much too large, while it extended an eighth of an inch under the gum all around. That is indeed heroic treatment. In crown- and bridge-work to-day, this illustration is not an exception, but one of the average operations.

*Dr. Warren.*—I agree with Dr. Jamison that judgment is essential. As far as plastics and gold crowns are concerned, both are to be used. All of us see crowns and bridges which should never have been placed in the mouth; but, at the same time, it is not advisable for us to speak of bridge-work and crown-work so as to make the impression that it is all bad. There are instances where a crown is the only means of saving a tooth. There are times when we find good firm roots where bridges can be placed to advantage. I have put fourteen teeth on a gold bridge (or band), anchored to two molars and two cuspids, and I have known such bridges to give excellent results. It is not advisable, as a rule, to go to extremes in any line of work, but as far as crown- and bridge-work is concerned, if it is properly done, much permanent good will ensue to the patient. As Dr. Jamison said, judgment is the all-important factor.

*Dr. Broomell.*—I reverted in my mind to the beginning of my practice when Dr. Kratzer spoke of Dr. Webb. And while he was certainly an extremist as to the matter of gold work, and while his fillings were excellent, there is no question but that he overdid the matter. There seemed to be what might be termed a filling craze, and everything must be filled with gold. I remember a case I had myself while at college. It was a bicuspid tooth, upon which I placed the rubber dam at nine o'clock in the morning and kept it on until four in the afternoon. When the work was finished, it was a very satisfactory operation. But that can now all be done away with by a perfect-fitting gold crown.

The essayist spoke of open-faced gold caps as being preferable in many cases, or probably in all cases, to the solid crown. I don't see how it is feasible to allow edges to remain unprotected which must continue intact if the secretions are not to disintegrate the cement underneath. I should think, under these conditions, it would only be a question of time when the crown would have to be removed.

*Dr. Kratzer.*—As to the remarks of Dr. Broomell in respect to open-faced crowns, I maintain that when it becomes necessary to remove them on account of leakage, we can still incise the root and put on a Richmond crown, having put off the final operation and gained valuable time.

*Dr. Jamison.*—I might say that I do not agree with Dr. Broomell as to open-faced crowns. I can cite a case where they gave me much satisfaction. A patient came to me with two bicuspids where the inner cusps had been broken; the teeth were still vital. By making an open-faced gold crown I was able to save those two teeth by showing very little gold at the cutting-edges, and the pulps were saved.

Dr. Kratzer made a good point when he said that, though an open-faced crown only lasted five or ten years, if you can save the superstructure five or ten years you are certainly in a good position to save the root for a number of years.

*Dr. Tees.*—My experience with open-faced crowns, as far as bridge-work is concerned, has been the same as Dr. Warren's. I have seen many put on, and with very good results, but if they are improperly constructed, they are worse than nothing.

Joseph Head, M.D., D.D.S.,
*Editor Odontological Society of Pennsylvania.*

